# A Challenging Diagnosis of Atypical Glut1-DS: A Case Report and Literature Review

**DOI:** 10.3389/fneur.2020.549331

**Published:** 2021-01-28

**Authors:** Miaomiao Yu, Jing Miao, Yudan Lv, Xue Wang, Wuqiong Zhang, Na Shao, Hongmei Meng

**Affiliations:** ^1^Department of Neurology and Neuroscience Center, First Hospital of Jilin University, Changchun, China; ^2^Beijing Friendship Hospital, Capital Medical University, Beijing, China; ^3^Weifang Traditional Chinese Medicine Hospital, Weifang, China

**Keywords:** intracranial infection, ketogenic diet, GLUT1-DS, paroxysmal non-kinesigenic dyskinesia, SLC2A1, paroxysmal exercise-induced dyskinesia

## Abstract

Glucose transporter type 1 deficiency syndrome (Glut1-DS) is a rare neurometabolic disorder caused by mutations of the SLC2A1 gene. Paroxysmal exercise-induced dyskinesia is regarded as a representative symptom of Glut1-DS. Paroxysmal non-kinesigenic dyskinesia is usually caused by aberrations of the MR1 and KCNMA1 genes, but it also appears in Glut1-DS. We herein document a patient with Glut1-DS who suffered first from paroxysmal exercise-induced dyskinesia and subsequently paroxysmal non-kinesigenic dyskinesia and experienced a recent worsening of symptoms accompanied with a low fever. The lumbar puncture result showed a decreased glucose concentration and increased white blood cell (WBC) count in cerebrospinal fluid (CSF). The exacerbated symptoms were initially suspected to be caused by intracranial infection due to a mild fever of <38.0°C, decreased CSF glucose, and increased CSF WBC count. However, the second lumbar puncture result indicated a decreased glucose concentration and normal WBC count in CSF with no anti-infective agents, and the patient's symptoms were not relieved apparently. The continuous low glucose concentration attracted our attention, and gene analysis was performed. According to the gene analysis result, the patient was diagnosed with Glut1-DS finally. This case indicates that the complex paroxysmal dyskinesia in Glut1-DS may be confusing and pose challenges for accurate diagnosis. Except intracranial infection, Glut1-DS should be considered as a differential diagnosis upon detection of a low CSF glucose concentration and dyskinesia. The case presented here may encourage clinicians to be mindful of this atypical manifestation of Glut1-DS in order to avoid misdiagnosis.

## Background

Glucose transporter type 1 deficiency syndrome (Glut1-DS) is a rare neurometabolic disorder caused mainly by *de novo* mutations of the SLC2A1 gene (1). SLC2A1 encodes the glucose transporter type 1, a membrane protein that transports glucose across the blood–brain barrier. Though mostly *de novo*, mutations of SLC2A1 gene have also been reported with an autosomal dominant mode of inheritance in rare families (2). The classical phenotype includes early-onset epilepsy, motor and mental retardation, and acquired microcephaly. Non-classical phenotypes include paroxysmal exertion-induced dyskinesia (PED), absence epilepsies, particularly early-onset absence epilepsy and childhood absence epilepsy, myoclonic astatic epilepsy, episodic choreoathetosis and spasticity, and focal epilepsy (3). We herein report a case of Glut1-DS with changing, complex paroxysmal dyskinesias (PDs), in which the worsened dyskinesia was initially covered by intracranial infection. The patient was ultimately diagnosed with Glut1-DS according to the low cerebrospinal fluid (CSF)-to-blood glucose concentration ratio and gene analysis result after 21 years of the onset.

## Case Presentation

**Figure 1:**
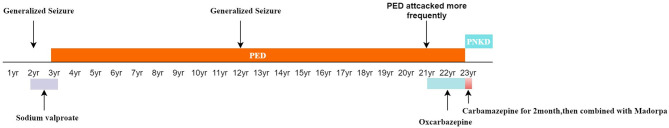
The timeline of the patient's symptom.

A 23-year-old woman presented to our hospital with involuntary movement attacks, which she had exhibited for about 20 years. The patient has an unremarkable family history, and her antenatal and perinatal histories and postnatal development were uneventful. At the age of 2, she underwent her first non-febrile generalized seizure. She was administered sodium valproate for 1 year. While another seizure occurred at the age of 12, the patient had since remained seizure-free. At the age of 3 years, involuntary movement attacks without impairment of consciousness began to occur after prolonged exercise or fasting. Attacks were characterized by intermittent dystonia of right unilateral limbs, manifesting as elbow joint, wrist joint, knee joint alternating flexion, twist, and adduction, especially the upper limb. Left limbs were involved occasionally. The involuntary movements lasted from 3 to 5 min. The episodes occurred only one or two times every month. She was diagnosed with dystonia, but no drugs were prescribed. The attacks often occurred before menstruation since her adolescence. At the age of 21 years, the pattern of attacks did not change, but it occurred more frequently than before, about once a week. The patient received a prescription for oxcarbazepine; while the drug helped to control the symptoms at first, it gradually became unhelpful. Four months before admission, the patient's manifestations became less associated with exercise or fasting, and the frequency increased to two to three times weekly. Oxcarbazepine was replaced with carbamazepine. Two months before admission, the symptom worsened after a trip to the seaside: the patient experienced continuous attacks, presenting as involuntary movements of the right upper and lower limbs, with affected limbs becoming painful. The degree of movement was similar to previous attacks; however, the episodes lasted from 3 to 30 min with intervals of 15–20 min; carbamazepine combined with Madopar could not relieve her PDs. Her head circumference was normal. Psychomotor development did not show any abnormalities according to her parents. The timeline of the patient's symptom is shown in Figure 1.

Neurological examination revealed positive bilateral Hoffman signs and decreased muscle tone. Cranial nerves, motor strength, and sensory examination were normal. Brain and spine magnetic resonance imaging, lung computed tomography, and laboratory blood exams (including the assessments of ceruloplasmin and erythrocyte sedimentation rates) were unremarkable.

**Figure 2:**
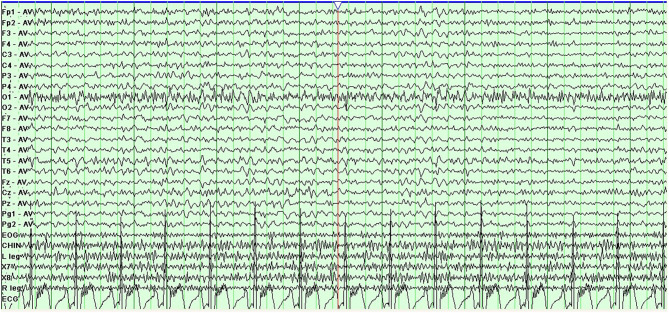
Electroencephalogram shows sharp-slow and delta discharges; bilateral frontal area.

Prolonged electroencephalogram monitoring showed sharp-slow and delta waves in the bilateral frontal area (Figure 2). Considering the worsening of her symptoms after her travels and a low fever of under 38.0°C, which had begun 4 days before admission to our hospital, we supposed that the worsened dyskinesia could be attributed to infectious factors. After written informed consent was obtained from the patient, lumbar puncture was performed, which showed a white blood cell (WBC) count of 20 × 10^6^/L and a glucose concentration of 1.9 mmol/L (reference value: 2.3–4.1 mmol/L) in CSF; before the lumbar puncture, the patient's blood glucose concentration was 4.39 mmol/L. The CSF:blood glucose ratio was 0.43 (normal range: 0.62–0.68) [3]. These findings informed a speculative diagnosis of intracranial infection. However, antibody testing for *M. tuberculosis* and viruses in the blood and smear tests for *M. tuberculosis* and *Cryptococcus* in CSF showed negative results. A second lumbar puncture was performed 6 days later, showing a WBC of 2 × 10^6^/L and a glucose concentration of 2.0 mmol/L in CSF. Tested the same morning, the patient's blood glucose concentration was 4.50 mmol/L; the ratio of CSF to blood glucose concentration was 0.44. Though the WBC in the CSF decreased to normal levels, the patient's symptoms did not abate fully. Her symptoms, medical history, and persistent reduced CSF:blood glucose ratios together suggested a neurometabolic disorder. We therefore subjected the patient and her parents to genetic analyses and identified a heterozygous point mutation (c.940G>A) in exon 7 of the SLC2A1 gene, which causes a substitution of amino acid 314 from glycine to serine (p.Gly314Ser). The mutation was not found in her parents. Ultimately, the genetic analysis informed a diagnosis of Glut1-DS. The patient began a ketogenic diet (KD), and the PDs disappeared within 3 months.

## Discussion

We herein presented the case of a female patient who initially suffered from PED and subsequently from PNKD, with self-limiting generalized seizures. She was diagnosed with dystonia once and the worsened symptom was considered to be due to intracranial infection. Finally, Glut1-DS was diagnosed through genetic analysis at the age of 23.

Initially, we did not suspect the diagnosis of her dystonia. Considering the patient's increased WBC, decreased glucose concentration in the CSF, and fever, we thought the worsened PDs was triggered by an intracranial infection. The result of the second lumbar puncture showed normal WBC in CSF; however, her symptoms did not recede fully, and our initial suspicion was thus ruled out. We supposed that slight intracranial infection was the cause of increased WBC in CSF, but not the main cause of continuous decreased glucose concentration and worsened PDs. She was almost misdiagnosed. The inaccurate history misled us and we had to reconsider her condition. The persistent hypoglycorrhachia indicated a metabolic disease, and the gene analysis results suggested a diagnosis of Glut1-DS. While molecular analysis of the SLC2A1 gene is the basis of the diagnosis, low CSF glucose levels (in the absence of meningitis) and a low CSF to blood glucose concentration ratio (<0.45) are the best biochemical support for diagnosis (1). The mutation (c.940G>A) in exon 7 of the SLC2A1 gene causes the amino acid 314 to change from glycine to serine (p.Gly314Ser) on transmembrane segment 8, which disrupts protein function. The mutation has been reported in both familial and sporadic cases, in which patients presented with PED accompanied by various kinds of seizures (4, 5).

**Figure 3:**
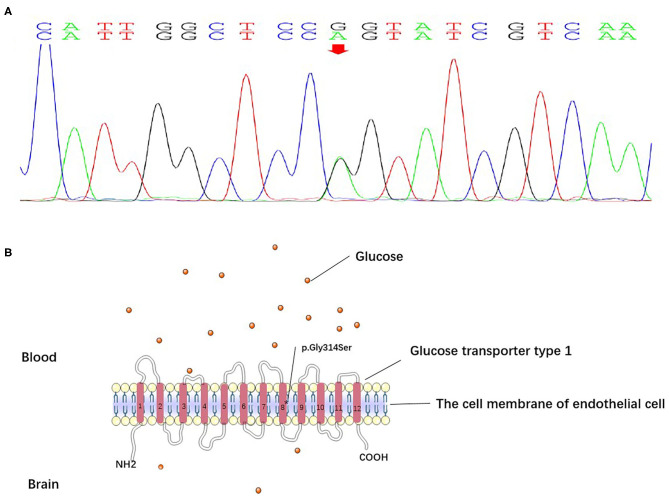
**(A)** The DNA sequencing results of the patient. The results suggested a heterozygous point mutation (c.940G>A) in exon 7 of the SLC2A1 gene. **(B)** Glucose transporter type 1 is composed of 12 transmembrane segments with N- and C-terminals. The mutation (c.940G>A) in exon 7 of the SLC2A1 gene caused the substitution of glycine 314 with serine (p.Gly314Ser) on transmembrane segment 8, which disrupted protein function.

Glut1-DS is characterized by normal serum glucose and hypoglycorrhachia, which results in seizure, developmental delays, movement disorders, and other complex manifestations. Glucose, the main energy source of the mammalian brain, is delivered by Glut1 to astrocytes and neurons via the blood–brain barrier (6). Mutations of SLC2A1, localized to chromosome 1p34.2 and encoding Glut1, cause a defect in glucose transport by influencing the function of Glut1 (Figure 3) (7). The most suitable therapy for Glut1-DS is a KD, a high-fat, low-carbohydrate diet, which replaces glucose with ketone bodies as fuel to the brain. KD should be commenced as early as possible because prompt initiation of the diet may prevent seizures, movement disorders, and cognitive impairment (8).

Glut1-DS is difficult to be recognized and distinguished from other diseases, timely and easy to be misdiagnosed due to its complex and wide clinical spectrum. In a study performed by M. Hully et al., the results indicated that the median age of onset is 7.5 months, while the median age of diagnosis is 8 years 5 months (9). Considering the positive response to KD, diagnosing Glut1-DS as soon as possible is particularly important. Of all manifestations, epilepsy is the most common feature, especially the early-onset seizures (3). Most patients with Glut1-DS develop movement disorders, including gait disturbance, action limb dystonia, chorea, cerebellar action tremor, myoclonus, dyspraxia, non-epileptic paroxysmal events, and so on (10). Glut1-DS can also manifest kinds of atypical features. Ros-Castelló et al. reported that a woman who presented paroxysmal dystonic gait, early-onset epilepsy, and episodic migraine was once misdiagnosed as cerebral palsy and diagnosed as Glut1-DS at the age of 52 years old (11). Another study suggested that some patients with Glut1-DS could be considered as having pure or complex hereditary spastic paraplegia (HSP) (12). It indicated that genetic analysis for SLC2A1 should be taken into consideration for pediatric-onset HSP patients, especially those without pathogenic variants in HPS gene (12). Paroxysmal eye–head movements, paroxysmal hemiparesis, dysarthria, or aphasia also occur in Glut1-DS patients, which would lead to diagnostic errors (13, 14). Brain MRI images of most Glut1-DS patients are normal. A research by Akasaka et al. revealed that magnetic resonance spectroscopy imaging of Glut1-DS patients showed higher glutamate–glutamine complex/creatine ratio in thalamus compared to normal population, which may assist diagnosis of Glut1-DS (15). Leed et al. developed a flow chart for the differential diagnosis of neurologic disorders from low CSF glucose levels in children and young adults. The first step is to identify whether the CSF/blood glucose ratio is >0.6; a normal CSF/blood glucose ratio indicates hypoglycemia to be the cause of hypoglycorrhachia. Next, low CSF glucose levels combined with an elevated cell count, CSF protein, and CSF lactate may indicate CSF infection, CSF inflammation, leptomeningeal metastasis, mitochondrial disorders, or even ischemia and hypoxia. Low CSF glucose also presents in cases of post-hemorrhagic hydrocephalus along with an increased erythrocyte count and/or bilirubin (16). When treating a patient with early-onset, drug-resistant epilepsy, movement disorders, or motor and mental retardation, especially with isolated hypoglycorrhachia, Glut1-DS should be suspected, and genetic analysis should be performed (17).

In conclusion, many patients with Glut1-DS remain undiagnosed or misdiagnosed on account of the disorder's varied phenotype. A detailed medical history is therefore needed for diagnosis. In addition, distinguishing suggestive symptoms from complex symptoms is essential. This case indicates that the complex PDs in Glut1-DS may be confusing and difficult to distinguish. Except intracranial infection, Glut1-DS should be considered as a differential diagnosis upon detection of a low CSF glucose concentration and dyskinesia. Clinicians should be aware of this atypical presentation of Glut1-DS in order to avoid misdiagnosis. A complete understanding of Glut1-DS will help to facilitate its diagnosis as well as its effective treatment with KD (13).

## Ethics Statement

Written informed consent was obtained from the individual(s) for the publication of any potentially identifiable images or data included in this article.

## Author Contributions

HM contributed to the conception and design of the paper. MY and JM wrote the first draft of the manuscript. YL, XW, NS, and WZ wrote sections of the manuscript. All authors contributed to manuscript revision, and have read and approved the submitted version.

## Conflict of Interest

The authors declare that the research was conducted in the absence of any commercial or financial relationships that could be construed as a potential conflict of interest.
